# COVID-19 Vaccine Hesitancy: The Role of Socioeconomic Factors and Spatial Effects

**DOI:** 10.3390/vaccines10030352

**Published:** 2022-02-24

**Authors:** Jim Lee, Yuxia Huang

**Affiliations:** 1 College of Business, Texas A&M University–Corpus Christi, Corpus Christi, TX 78412, USA; 2 School of Engineering & Computing Sciences, Texas A&M University–Corpus Christi, Corpus Christi, TX 78412, USA; lucy.huang@tamucc.edu

**Keywords:** COVID-19, vaccine hesitancy, socioeconomic and demographic factors, spatial spillover

## Abstract

This paper investigates the spatial dimension of socioeconomic and demographic factors behind COVID-19 vaccine hesitancy. With a focus on a county with considerable sociodemographic diversity in the state of Texas, USA, we apply regression models to census-tract-level data of the unvaccinated population. In addition to disparities in accessing the vaccination service, particularly for residents in rural areas, empirical results confirm under-vaccination among lower socioeconomic neighborhoods and communities with signs of distrust in government. The spatial model regressions further underscore the impact that vaccine hesitancy among residents in one community spread to its nearby communities. This observed spatial spillover effect is attributable to the geographic interactions of similar socioeconomic groups.

## 1. Introduction

The newly developed COVID-19 vaccines have been widely viewed in the United States as effective at preventing the coronavirus disease, hospitalization, and even deaths. Following the U.S. Food and Drug Administration’s (FDA) full approval of the Pfizer-BioNTech vaccine (Pfizer, New York, NY, USA) in August 2021, the number of vaccinations surged across the nation [1].

Still, despite the widely publicized benefits and availability of the COVID-19 vaccination, no more than 60 percent of the U.S. population was fully vaccinated by the end of 2021 [2]. Public health experts and policymakers alike have struggled to identify strategies to continue to reduce COVID-19 vaccine hesitancy, defined as the delay in acceptance or refusal of the service [3].

To enhance the progress of COVID-19 vaccination, the critical first step is to understand the underlying drivers behind unvaccinated individuals, particularly their hesitancy or skepticism about the service. Much of the fast-growing body of research on this topic emphasizes disparities in different sociodemographic groups’ attitudes or fears towards the available vaccines (e.g., [4,5,6,7,8,9]). It remains a challenge to effectively implement vaccine campaign strategies to target those population segments with under-vaccination rates.

This paper fills this knowledge gap by offering a geographic or spatial context to the socioeconomic and demographic factors behind vaccination hesitancy. Socio-demographics tend to cluster in various neighborhoods of our study area. The spatial patterns that we identify will potentially help public health officials and city planners improve the vaccination rates of underserved neighborhoods and beyond.

## 2. Literature Review

Most studies on the sources of unwillingness to vaccinate draw inferences from cross-sectional survey data. The consensus points to a higher hesitancy among the less-educated, females, racial/ethnic minorities, people living in rural areas, and those with lower income or economic security [6,7,8,9,10,11,12,13]. In the United States, the economically disadvantaged population is predominantly Hispanic or Black. These racial/ethnic minority groups are also more likely to work in high-contact occupations, such as hotel housekeeping and restaurant food service, which are particularly vulnerable to the infection risk of a virus contagion [14].

However, most studies that focus on vaccine hesitancy during the COVID-19 pandemic lack a geospatial perspective, which has played a crucial role in understanding how the pandemic has unfolded over space and time. Because the spread of infectious diseases akin to the coronavirus is inherently a spatial process primarily through close human contact, geospatial-oriented data are indispensable for public health officials and scientists to monitor, model, and control the spread of COVID-19 [15]. Similarly, Sarkar and Morshed [16] show that as a result of “spatial connectivity” within a broad region, a vaccine rollout strategy, especially in the face of limited vaccine supply, should correspond to identified spatial patterns across different socioeconomic communities.

The transmission risk of COVID-19 tends to be higher in indoor spaces and close quarters, such as prisons and nursing homes [17], and densely populated neighborhoods, such as city centers [14]. Wong and Li [14] also found spillovers in virus outbreaks across U.S. counties. Unlike the risk of infection, however, there is little research to understand spatial patterns of vaccination rates in response to COVID-19 outbreaks.

Instead, the existing literature highlights the influence of subjective perceptions and psychological factors on vaccine acceptance. People are more willing to get vaccinated if they have more infection- or health-related fears, or if they trust health officials’ dissemination of COVID-19-related information rather than relying on the information on social media [4,5,10]. Reich [18] found that parents with high levels of social privilege are more likely to refuse vaccines for their children. They also tend to be geographically clustered or socially connected to provide information and emotional support for each other within a social group.

In the United States, citizens’ political views, or ideological allegiance in general, also seem to have a strong influence on their willingness to receive COVID-19 vaccines. During the early months of the nationwide vaccination program, Republican voters, and some minority groups, notably Hispanics and Blacks, lagged in receiving shots [19,20]. While the racial gaps narrowed through the rest of 2021, the gap between Republicans and Democrats remained wide. A recent survey indicated that nearly 40% of Republicans were unvaccinated in October, compared with about 10% among Democrats [12].

Political affiliation in the U.S. is seemingly tied to demographics. For instance, during the 2020 presidential election, White men and people living in rural communities tended to favor the Republican party or Donald Trump; voters for the Democratic party or Joe Biden were likely Black women and urban dwellers [21]. Political polarization in vaccination rates appears to be uniquely a U.S. phenomenon, reflecting citizens’ perceptions of government measures and information [3].

To summarize, the willingness to receive vaccination, particularly against COVID-19, is likely subject to the confluence of social, economic, demographic, and some subjective factors. From this perspective, intervention strategies to improve the success of vaccination programs should take such determinants into account. This paper contributes to a better understanding of the drivers of vaccine acceptance by exploring spatial variations in these determinants. Neighborhood spillovers have been found to affect people’s exposure to COVID-19 infection [14,22].

Beyond exploring a wide array of socioeconomic and demographic characteristics, we consider the spatial dimension of vaccinations within a U.S. community. To do so, we turn to a novel dataset of residents who have taken COVID-19 vaccines in one county with diverse demographics. We find that under-vaccination is not evenly distributed across the neighborhoods of this region. The finding of spatial clustering patterns in vaccine hesitancy and the associated socioeconomic characteristics would help the public efforts to successfully promote universal vaccination acceptance.

## 3. Methods and Materials

### 3.1. Study Area

The geographic area of our study is Nueces County in Texas, a state that has experienced remarkable waves of COVID-19 outbreaks since the beginning of 2020. Counties and their equivalents are the primary administrative or political units in the U.S. According to U.S. Census data, Nueces County’s population in 2020 was roughly 353,000. Ranked at the 97th percentile among U.S. counties in population size, the area owes much of its relatively large population to the city of Corpus Christi it encompasses. Corpus Christi accounts for approximately 90% of Nueces County’s population but slightly less than 20% of its land area.

In contrast to the rest of the nation, Hispanics make up 65% of Nueces County’s population, making this ethnic group a majority instead of a minority nationwide. The adult obesity rate is 33.2% and the diabetes rate is 13% [23]. By October 2021, about 60% of its residents 12 years and older were fully vaccinated. The geographic and demographic characteristics make this county an interesting case for studying the determinants of under-vaccination.

The Corpus Christi–Nueces County Public Health District is the primary authority that provides the COVID-19 vaccination service to the public within Nueces County. The Pfizer-BioNTech and Moderna vaccines require two doses with the second dose about three weeks after the first. The Johnson and Johnson vaccine was first available in March 2021, and it required one dose instead of two.

In December 2020, the public health district began its COVID-19 vaccination service to the public at the county’s Richard M. Borchard Regional Fairgrounds next to the city of Corpus Christi. Online preregistration was required for the drive-thru vaccination service at that site and seniors were given priority. The Moderna vaccine was the only option before Pfizer became available in early February.

Throughout 2021, the public vaccination service expanded with additional locations across the county, such as a major drive-thru facility at the American Bank Center in Corpus Christi downtown. By October, the public health district had operated 18 sites, including walk-thru sites such as the one in the area’s largest shopping mall, La Palmera. Including other outlets, such as pharmacies, health clinics, and hospitals, the number of vaccination sites across the county had grown to 104.

In late October, the public health district announced its availability of booster shots for residents 18 years or older. For this reason, the window of our data collection spans between January and October 2021. Given our focus on the spatial perspective of the vaccinated population, the vaccination records from the public health district are grouped into Nueces County’s census tracts.

One advantage of focusing on one instead of multiple counties is that all residents in Nueces County should have received the same local public health information and announcements. In addition to daily updates on local COVID-19 cases, hospitalized patients, and deaths released by the public health district, all residents were subjected to the same countywide policy measures that also covered the city of Corpus Christi, such as business lockdown orders in early 2020 and the subsequent stepwise business reopening announcements. This allows us to control the potential effects of otherwise different local government policy actions and public information.

Most residents should also have the same opportunity to receive the COVID-19 vaccination service provided locally to the public. Nevertheless, the vaccination rates of different population groups might differ due to their own inherent characteristics and behaviors, including the influence of social networks [18] and access to the vaccine sites [24].

To our knowledge, this is the first study of vaccination hesitancy with official records as opposed to survey data. Surveys are well known to suffer from potential selection or response bias as there is no reliable way to ensure that respondents reveal their true preferences. Even widely followed surveys by the U.S. Census and Delphi-Facebook have been found to substantially overestimate COVID-19 vaccine uptakes in 2021 [25]. On the other hand, the official vaccination records lack individuals’ data beyond their basic demographic information.

To circumvent the lack of some socioeconomic data of our interest, such as income and educational attainment, we focus on census-tract-level vaccination data. By construction, a census tract typically contains 4000 people, so its land area varies depending on population density. The U.S. Census Bureau considers a census tract the best description of a “neighborhood.”

### 3.2. Vaccination Determinants

The objective of our empirical work is to evaluate the sources of under-vaccination during the COVID-19 pandemic. In light of the existing literature that emphasizes the role of socioeconomic and demographic factors [6,7,8,10,11,12,13], we consider the various components that make up the U.S. Centers for Disease Control and Prevention’s [26] Social Vulnerability Index (SVI). The SVI is aimed at helping public health officials and policymakers better prepare for disease outbreaks and other disasters.

For each census tract, the CDC reports the census data for 15 social vulnerability metrics, such as poverty and lack of vehicle access. We apply their most updated dataset, which derives from the 2014–2018 Census estimates. Although these data are not for 2021, their cross-sectional patterns across different census tracts should not have changed meaningfully. The census-tract-level data in our study area are incomplete for four of the 15 SVI factors, all of which are measures of housing types, such as mobile homes. For this reason, we exclude these four housing factors. The other 11 metrics are grouped into the following four themes: socioeconomic status (the poverty rate, the unemployment rate, per capita income, and the population that has not finished high school), household composition and disability (population share age 65 and older, population share age 17 and younger, people with disabilities, single parents, and people who speak limited English), minority status (share of race/ethnic minorities), and transportation (households with no vehicle).

We consider those social vulnerability characteristics as primary sociodemographic determinants in our regression models presented below. Motivated by other studies (e.g., [8]), the models also control for the distinction between an urban and rural environment, which is measured by the population density (persons per square mile) of the census tracts.

In light of recent studies (e.g., [2]) that emphasize the role of partisanship in vaccinations across the U.S., we also consider the influence of people’s political views. To this end, we obtained data for the share of residents voting for Biden versus Trump during the 2020 presidential election. The data are drawn from the New York Times [27], which reported the numbers of votes for Biden and Trump at the local level. The data are the percentage of lead, or margin, for Biden. A negative value represents a lead for Trump.

### 3.3. Model Regression Methods

In our empirical work, we first apply the census tract data to ordinary least squares (OLS) regressions. Given the spatial dimension of our data, we further explore spatial patterns in vaccinations. Motivated by strong evidence of spatial clustering in the vaccination data as shown in Section 4 below, we consider the spatial autoregressive (SAR) approach [28]. The most general specification is the spatial Durbin model with K explanatory variables and N census tracts, as follows:

(1) y=ρWy+Xβ+WXθ+ε

where *y* is the N-vector of the unvaccinated population share; ρ is the spatial autocorrelation coefficient; Wy is a spatially lagged dependent variable where W is an N × N spatial weight matrix; *X* is an N × K matrix of explanatory variables so that WX is the corresponding matrix of spatially lagged explanatory variables; β  and θ are K-vectors of coefficients; ε is an N-vector of residuals. The spatial Durbin model nests the following two different models: (a) OLS if ρ = 0 and θ = 0; (b) SAR if θ = 0.

The spatial weight matrix *W* captures the connections between different census tracts. A popular way of parameterizing W is in the form of a first-order contiguity matrix, in which the spatial weights *w*_ij_ are non-zero when census tracts *i* and *j* share a border, and zero otherwise. The spatial weight matrix allows us to estimate for each of the following explanatory variable two types of effects: (a) the “local” or direct effects, which measure the impact of the explanatory variable of census tract *i* on the unvaccinated population share in census tract *i*; (b) the “spillover” or indirect effects captured by the spatial lags, which measure the impact of that variable of the neighboring census tracts on the unvaccinated population share in census tract *i*.

## 4. Empirical Results

### 4.1. Vaccination Data

For our empirical analysis, we first compiled data of vaccination records by the 78 census tracts within Nueces County. Excluding the five tracts with very few residents and no vaccination records, our analysis draws on 73 census tracts that contain data. The dependent variable is the share of the unvaccinated population in each census tract, in the sense that those residents had not received at least one shot of any of the COVID-19 vaccines, by October 2021. Our dataset is limited to those aged 12 and older, as the vaccines were not authorized for children younger than 12 years until later that year. We obtained data on the eligible population in each census tract from the 2020 Census.

**Figure 1:**
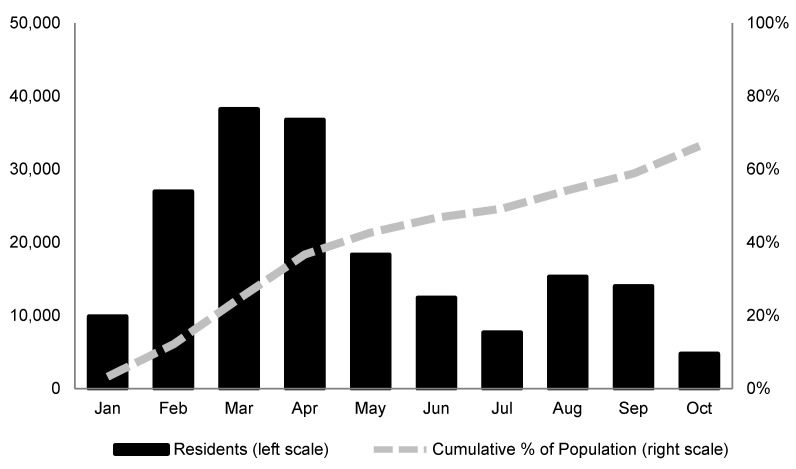
Fully vaccinated population in Nueces County, age 12 and older.

Figure 1 shows the number of fully vaccinated residents in Nueces County from January through October 2021. Those who had received two shots of the Pfizer and Moderna vaccines and one shot of Johnson and Johnson were considered fully vaccinated. The number of vaccine doses administered in Nueces County peaked in March.

After falling over the following four consecutive months, vaccinations surged again in the wake of the FDA’s approval of the Pfizer vaccine. By October, about 60% of the population age 12 and older were fully vaccinated and 9% had received only one dose of the Pfizer or Moderna vaccine.

**Figure 2:**
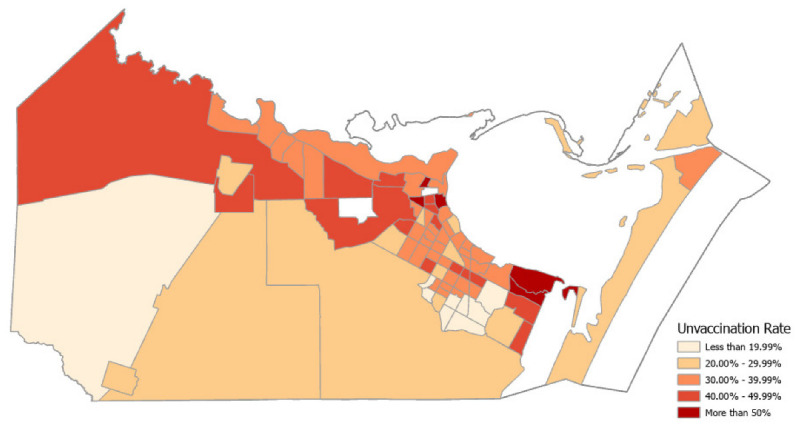
Percentage of unvaccinated population by census tract, age 12 and older.

Similar to the demographics, the vaccination rate varied remarkably across different neighborhoods within Nueces County. Figure 2 shows a map of the distribution by census tracts of the share of the unvaccinated population age 12 and older in October. The census tracts with the highest shares of unvaccinated people (over 40%) are mostly near the city of Corpus Christi’s downtown, followed by rural communities on the northwest side of the county. The census tracts with less than 20% of their unvaccinated population are primarily on the south side of Corpus Christi. Overall, the map reveals a pattern of clustering among census tracts in the sense that one census tract’s progress of vaccination is similar to its neighboring census tracts.

**Figure 3:**
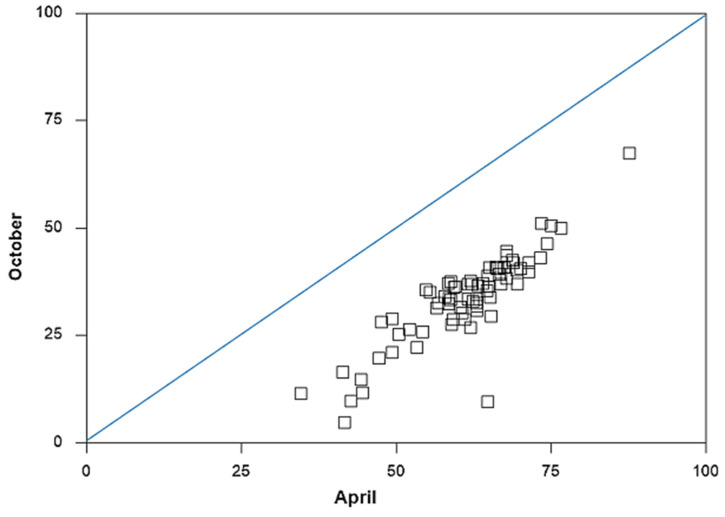
Unvaccinated population shares by census tract, April vs. October 2021.

Despite the observed spatial patterns across census tracts, the challenge of getting eligible individuals vaccinated seemed to prevail over time. To understand this, we compare the vaccination rates in October with those in April. Across the county, 28% more residents became fully vaccinated between May and October. Figure 3 displays a scatter plot of the full vaccination rates for census tracts between April and October. Clearly, the vaccination rate increased in tandem across most census tracts. This means little catchup over time from census tracts with relatively fewer vaccinations.

Census tract 59.00 was the only exception. The share of the unvaccinated population in this rural community near the city of Robstown reduced from 65% in April to slightly below 10% in October. Besides this outlier, evidence of strong persistence in the geographic patterns of vaccinations over time motivates our investigation of their determinants. Since our model controls for residents’ access to the vaccination sites through vehicle ownership and a proxy for the rural environment, our regression results shed light on vaccine hesitancy for the unvaccinated people.

### 4.2. Descriptive Statistics of Vaccination Determinants

Table 1 presents the descriptive statistics of the variables in our regression models. The dependent variable is the share of the population age 12 and older that remains unvaccinated. As the first row of the table shows, the average across the 73 census tracts is roughly 34%. The range of the unvaccinated population’s shares between 4.74% and 67.39% is remarkable.

The rest of Table 1 displays the descriptive statistics for the explanatory variables. Except for the measures of per capita income, political preferences (Biden voters), and population density, all variables are expressed as percentages of the census tract population. Accordingly, the typical census tract had about 14% of residents living in poverty, 15% without finishing high school, and 2% with limited English proficiency. About 13% of its residents were seniors aged 65 and older, about the same percentage with disabilities, about double the percentage of 17 years or younger, and 11% were single parents. The minority population across Nueces County was predominately Hispanic. Including Blacks, Asians, and American Indians, who together accounted for less than 8% of the local population, the racial/ethnic minority made up 73% of the local population.

The socioeconomic disparities are striking across the census tracts in Nueces County. For instance, the per capita income level ranged from $11,384 to more than five times that at $60,802. Likewise, more than half of the population had not finished high school in one census tract in the Westside district next to downtown Corpus Christi, while nearly none in another district on North Padre Island at the other end of the city. The wide range of voter shares for Biden (Democrats) versus Trump (Republicans) also reflected the extent of political polarization across different neighborhoods.

The spatial distribution of unvaccinated residents across Nueces County appears to be associated with the spatial patterns of different socioeconomic and demographic groups. Overall, residents were less likely to have received vaccinations if they lived in an economically disadvantaged neighborhood with lower income and educational attainment. For instance, the census tract 5.00 with the highest share of the unvaccinated population, at 67%, was at the upper end of downtown Corpus Christi. This is widely referred to as a “historically Black” community, with 93% of its population being ethnic minorities.

On the other extreme, almost 95% of the residents in the census tract 54.17 were vaccinated. This newly developed neighborhood on the south side of Corpus Christi had a poverty rate of less than 4%. The area also had the most educated residents, and less than 2% of them had not finished high school.

In our regression analysis, all variables are expressed in logarithmic terms to mitigate potential nonlinearity in the data. The measure of political preferences allows for negative entries in the case of a lead for Trump as opposed to Biden. Following a popular approach to dealing with negative values in a variable x, the log transformation applies to x + 1—minimum(x). The minimum value is −55, so the transformed data are the logarithm of the original values plus 56. Most of the explanatory variables that draw from the CDC’s SVI seem well-suited for our study, but it is obvious that some of those social vulnerability factors are highly correlated. For instance, census tract 56.02 had both the highest poverty and unemployment rates, while census tract 62.0 had both the lowest minority population and residents without finishing high school.

To understand the extent of correlation between the explanatory variables, Table 2 presents their pairwise Pearson correlation coefficients. The absolute size of a correlation coefficient larger than 0.5 suggests a strong relationship between two variables, and a negative value indicates an opposite relationship. According to the table, the per capita income of a census tract is highly correlated with all other socioeconomic variables, such as minority status, limited English proficiency, less than a high school education, and no vehicle ownership.

As expected, a higher income level is associated with measures of lower socioeconomic status. Similar to income, poverty is strongly associated with schooling, disabilities, and vehicle ownership. Finally, census tracts with relatively more residents voting for Biden as the U.S. president are neighborhoods with more minorities and non-English speakers. A strong correlation between the explanatory variables in a model can potentially increase the variance of the regression coefficients. We deal with the observed multicollinearity in the regression analysis in the next subsection.

### 4.3. Ordinary Least Squares Regressions

Table 3 displays the OLS results of three alternative model specifications. The dependent variable is the logarithmic value of the percentage of the population age 12 and older that remained unvaccinated by October. Column (1) lists the coefficient estimates of the baseline model with 13 explanatory variables. The 95% confidence intervals (c.i.) are listed in brackets next to the corresponding coefficient estimates. Except for the Biden voters and population density variables, those variables are components of the CDC’s SVI, representing socioeconomic and minority status, household composition, and access to transportation. The estimation results suggest that under-vaccination was more of a challenge for census tracts with a lower socioeconomic status, as represented by a higher poverty rate, lower per capita income, lower educational attainment, and lower vehicle ownership. The unemployment rate, however, does not offer the same qualitatively meaningful explanatory power as the other socioeconomic variables.

Contrary to the findings in other studies (e.g., [3,6,8]), the unvaccinated population share tended to be lower in census tracts with relatively more seniors. This seemingly odd finding might be attributable to the local efforts aiming to help inoculate homebound residents in Corpus Christi. The city has developed the Save Our Seniors (SOS) Homebound Program, in which firefighters provide in-home vaccinations for senior citizens beginning in February [29]. The program has served as a model for a statewide SOS initiative to identify and vaccinate homebound seniors in different neighborhoods across Texas [30].

The estimate for the political preference variable (Biden voters) supports the conventional wisdom that the vaccination rate was higher in neighborhoods with relatively more Democratic voters [2,20]. Also, the estimate of population density confirms higher vaccine hesitancy among people living in rural communities.

The estimation results of the baseline model should nevertheless be interpreted with caution. One primary shortcoming arises from the selection of explanatory variables. Even though most socioeconomic variables reflect different aspects of social vulnerability, some of them are highly correlated, as discussed in Section 3 above. For instance, per capita income and poverty are inherently associated with educational attainment as measured by high school graduation, English proficiency, and disabilities. Multicollinearity among these variables can potentially lead to bias in the variances of the coefficient estimates. In addition to the correlation matrix displayed in Table 2, the variance inflation factors (VIF) listed next to the coefficient estimates shed light on the potential effects due to multicollinearity. The poverty, income, and educational attainment variables have a VIF close to or higher than five, suggesting their strong correlations with other explanatory variables in the model.

To explore the potential multicollinearity problem, we also present regression results with alternative model specifications without those variables with relatively high VIFs. Column (2) shows the coefficient estimates without per capita income. In this case, the share of minorities becomes statistically significant. The negative sign indicates that minority-dominant neighborhoods tended to witness higher vaccination rates. This result contradicts previous findings in the literature (e.g., [6,8]) that emphasize vaccine hesitancy among racial minorities and other economically disadvantaged populations. This surprising result can be attributed to the fact that Hispanics were more likely to be Democrat as opposed to Republican voters, especially in Nueces County.

Column (3) shows the corresponding results without poverty and income. Most coefficient estimates of the remaining variables are nevertheless similar to those in column (2). The overall goodness of fit, as measured by R^2^ adjusted for the number of explanatory variables, also does not change. This perhaps reflects the strong relationship of the two excluded variables with educational attainment. Intuitively, residents’ educational attainment affects their income levels and their likelihood of falling into poverty.

### 4.4. Spatial Regression Results

A popular test for spatial dependence is Moran’s I statistic, which is 0.30 for the dependent variable. The test’s null hypothesis of spatial randomness (absence of spatial dependence) can be rejected at the 1% significance level. The positive Moran’s I statistic suggests clustering of similar data values across different census tracts. The Moran’s I statistics for the residuals of all the OLS model regressions in Table 3 are also statistically significant, suggesting the presence of spatial autocorrelation. For instance, the Moran’s I statistic for the residuals of the model in column (3) is 0.18, which is significant at the 1% level.

Supported by the above statistical evidence of spatial autocorrelation, Table 4 shows the model regression results of the alternative SAR and Durbin specifications. The set of explanatory variables corresponds to those listed in column (3) of Table 3. The first column lists the regression results of SAR, which augments the OLS model with a spatially lagged dependent term (Wy). The estimate for spatial autocorrelation (ρ) is 0.32, suggesting that nearly one-third of one census tract’s vaccine hesitancy was related to that in its neighboring census tracts. The coefficient estimates of most variables do not alter appreciably from their OLS counterparts, even when spatial autocorrelation is taken into account. One notable exception is the variable for seniors (age 65 and older), which becomes statistically significant at the 10% level.

As for the OLS model, the SAR model suggests that the vaccination rate was lower among residents without a vehicle or those living in a less-populated, rural community. Controlling for these two factors that likely affected people’s access to the COVID-19 vaccination sites, less-educated people and voters for the Republican party were more likely to refuse the COVID-19 vaccines.

Compared with OLS, the SAR model provides a better fit to the data, as supported by all the summary statistics (R^2^, log likelihood, and standard error). In addition, the Lagrange multiplier statistic for testing the spatial autoregressive term is 3.99, which is significant at the 5% level. Alternatively, a likelihood ratio (LR) test based on comparing the log likelihood values of the OLS and SAR models is 3.44, which is also significant at the 10% level.

Compared with SAR, the Durbin model specification also includes the spatial lags of the explanatory variables. The right panel of Table 4 shows the coefficient estimates for the explanatory variables and their spatial lag terms in two separate columns. The LR test for comparing the overall goodness of fit of the Durbin model against SAR is 17.44, which is also statistically significant at the 10% level. This supports the role of the spatial spillovers in socioeconomic factors for explaining vaccination hesitancy.

Alternatively, we can evaluate the validity of the spatial Durbin model versus the respective OLS and SAR model specifications through tests on the coefficient estimates. The chi-squared statistic for testing the null hypothesis of ρ = 0 and θ = 0 is 34.12, which is statistically significant at the 1% level. This supports the collective explanatory power of all spatial lag terms in the Durbin model. The corresponding test for the Durbin specification against SAR draws from testing the null hypothesis of θ = 0 (the coefficient of the spatial lags of the 11 explanatory variables). The chi-squared statistic at 23.79 is also statistically significant at the 1% level. Taken together, these test results confirm the validity of the spatial Durbin model over the OLS and SAR specifications as special cases. In other words, spatial autocorrelation prevails in both vaccine hesitancy and its determinants.

The coefficient estimates of the spatial Durbin model reveal several noteworthy findings. First, the inclusion of the spatially lagged explanatory variables more than doubles the estimate of the autoregressive coefficient (ρ) from 0.32 to 0.85, perhaps due to strong relationships between the spatial lags of dependent and explanatory variables. Second, the point estimates for the coefficients of the explanatory variables tend to be higher when spatial lags are added to the model, while their qualitative results remain largely the same. Finally, most spatial lags that are statistically meaningful are also positive in their point estimates. The positive spatial spillover effects implied by those estimates suggest that the socioeconomic status of a neighborhood is directly tied to its nearby neighborhoods.

## 5. Discussion

In addition to transportation barriers, particularly for residents living in rural communities, our regression results confirm the role of socioeconomic factors that reflect economic insecurity, particularly educational attainment, that also affects income earnings. These findings complement the existing literature that focuses on social inequity in COVID-19 vaccinations [6,7,8,9,11]. On the other hand, our empirical results contradict earlier studies that reported challenges facing racial/ethnic minorities in the U.S. [19,20]. Within the South Texas community from which we drew data, neighborhoods with a larger Hispanic population share tended to show higher vaccine acceptance than neighborhoods with a larger non-Hispanic White population share. The root cause behind this counterintuitive finding is difficult to pinpoint, but this might point to the extent of trust or distrust in the local government across different demographic groups within the same community.

Moreover, controlling for a myriad of sociodemographic factors, vaccine hesitancy also tends to be lower among the census tracts with relatively more voters for Biden in the 2020 presidential election. In line with recent observations [2], this finding highlights the impact of political polarization on public distrust in government information and actions. Hispanics also tend to favor the Democratic party, and minority-dominated neighborhoods witness higher vaccination rates.

Beyond the finding of socioeconomic factors that align with the earlier literature on the drivers of vaccine hesitancy during the COVID-19 pandemic, our empirical results highlight neighborhood disparities in vaccinations. The census tracts with more residents without a vehicle and rural communities tend to have lower vaccination rates. The spatial model regressions further suggest that vaccine hesitancy among residents in one neighborhood is directly related to vaccine hesitancy in its nearby neighborhoods. In line with the finding of spatial clustering in vaccine acceptance [9], the observed spatial spillover effect is attributable to the geographic interactions between similar socioeconomic groups.

COVID-19 has disproportionately affected ethnic minorities and other economically disadvantaged groups [14,31]. Yet we have presented evidence that people in lower socioeconomic neighborhoods have also stayed behind in receiving COVID-19 vaccinations, and those neighborhoods tend to cluster together. While what has driven the partisan gaps in vaccinations is unclear, our evidence lends itself to signs of disparate beliefs about vaccines, perceptions of public information, or distrust in government [3]. This sets the stage for Republican and Democratic politicians alike to improve vaccination outcomes by fostering a more inclusive community. Indeed, Pink et al. [32] found that unvaccinated Republicans were more willing to get vaccinated if they saw an endorsement from a prominent political figure, in this case, either Donald Trump or Joe Biden.

A consensus has emerged that getting the vast majority of people vaccinated is necessary to overcome the COVID-19 pandemic. Our empirical results together highlight some critical challenges facing U.S. policymakers and local public health officials in their efforts to vaccinate residents. While the geographical scope of this study allows us to evaluate the disparities across different neighborhoods, it remains unclear whether the findings from that south Texas county accurately represent other U.S. communities. From this perspective, one avenue for future research is to extend the focal area to other communities with different sociodemographic makeups. Another fruitful research focus is identifying the factors behind the observed political polarization in the unvaccinated population.

## 6. Conclusions

This paper aims to investigate the spatial dimension of socioeconomic and demographic factors behind COVID-19 vaccine hesitancy, as measured by the unvaccinated population. To this end, we investigated a novel dataset of vaccination records in Nueces County, which is a community in the state of Texas with an outsized minority population and diverse socioeconomic groups. The empirical results draw primarily on the set of social vulnerability factors reported by the CDC.

The vaccination rate was not evenly distributed across our study area. Neighborhoods with more Republicans and less-educated residents were more likely to refuse the COVID-19 vaccination. Vaccination also presented a challenge to those living in rural areas or without a vehicle. Overall, the wide-range of localized responses to the public COVID-19 vaccination service points to the drawback of offering a summary of the U.S., or even county-level, vaccination rates, because a regional average obscures disparate conditions across local neighborhoods.

Our study draws on official vaccination records as opposed to self-reported survey responses that the vast majority of the related literature relies on. Our findings, therefore, should be free from the survey sampling bias that has been found to plague even the largest surveys ever conducted for COVID-19 vaccination, such as those conducted by the Delphi-Facebook and Census Household Pulse [25]. Our regression analyses have further confirmed various sources of vaccination barriers and hesitancy, including educational attainment and political affiliation, which led those surveys to overestimate vaccine uptakes in the U.S. due to the under-representation of less vaccinated demographic groups.

Instead of one-size-fits-all policies, strategies to promote vaccination acceptance and to respond to future contagion outbreaks might benefit from prioritizing individual socioeconomic clusters. A good case in point is the city of Corpus Christi’s place-based SOS initiative, which is associated with higher vaccination rates among otherwise under-served neighborhoods with disproportionately more elderly and other homebound residents.

## Figures and Tables

**Table 1:** Summary statistics for variables by census tract.

Variable	Mean	Median	Std. Dev.	Maximum	Tract	Minimum	Tract
Unvaccinated Pop. (%)	33.66	35.50	10.43	67.39	5.00	4.74	54.17
Poverty (%)	16.22	14.40	9.72	46.60	56.02	3.20	54.09
Unemployment (%)	5.88	4.70	4.96	28.50	56.02	0.30	6.00
Per Capita Income ($)	27,029	24,702	10,715	60,802	62.00	11,384	56.02
No High School (%)	19.34	15.10	13.65	54.70	9.00	1.30	62.00
Age 65 and older (%)	13.86	13.20	4.40	29.30	64.00	3.40	54.13
Age 17 and younger (%)	25.33	26.00	5.34	37.50	17.01	2.50	64.00
Disabilities (%)	14.07	13.30	5.10	34.90	64.00	5.40	54.17
Single parents (%)	11.00	11.30	5.72	25.30	33.05	0.00	64.00
Minorities (%)	72.91	72.60	16.86	98.50	9.00	13.50	62.00
Limited English (%)	2.83	2.20	2.33	13.30	16.02	0.10	31.02
No vehicle (%)	8.17	5.30	8.29	41.50	64.00	41.50	14.00
Biden voters (%)	11.57	12.00	26.25	56.00	5.00	−55.00	58.02
Pop. density (persons/mi^2^)	3,870	4,357	2,518	9,123	33.05	17	60.00

**Table 2:** Correlation coefficients between explanatory variables.

	Variable	(1)	(2)	(3)	(4)	(5)	(6)	(7)	(8)	(9)	(10)	(11)	(12)	(13)
(1)	Poverty	1.00												
(2)	Unemployment	0.47	1.00											
(3)	Per Capita Income	−0.69	−0.48	1.00										
(4)	No High School	0.68	0.39	−0.77	1.00									
(5)	Age 65 and older	0.18	0.02	0.16	0.15	1.00								
(6)	Age 17 and younger	0.10	0.18	−0.34	0.13	−0.59	1.00							
(7)	Disabilities	0.60	0.24	−0.42	0.61	0.56	−0.29	1.00						
(8)	Single parents	0.60	0.28	−0.52	0.32	−0.30	0.50	0.10	1.00					
(9)	Minorities	0.59	0.46	−0.86	0.77	−0.12	0.31	0.33	0.40	1.00				
(10)	Limited English	0.47	0.23	−0.47	0.63	0.13	0.17	0.39	0.29	0.62	1.00			
(11)	No vehicle	0.66	0.08	−0.43	0.55	0.29	−0.21	0.64	0.27	0.32	0.30	1.00		
(12)	Biden voters	0.42	0.32	−0.56	0.67	−0.04	0.19	0.27	0.23	0.56	0.54	0.28	1.00	
(13)	Population density	0.27	−0.02	−0.20	0.12	−0.02	−0.13	−0.05	0.06	0.32	0.23	0.14	0.29	1.00

**Table 3:** OLS results.

Variable	(1)			(2)		(3)	
	Coeff.	95% c.i.	VIF	Coeff.	95% c.i.	Coeff.	95% c.i.
Constant	−1.21	(−7.58, 5.16)		5.31	(3.73, 6.89) *	5.21	(3.65, 6.77) *
Poverty	0.18	(0.02, 0.34) **	4.49	0.09	(−0.05, 0.23)		
Unemployment	0.01	(−0.16, 0.18)	1.58	−0.03	(−0.09, 0.03)	−0.02	(−0.07, 0.03)
Per Capita Income	0.53	(0.04, 1.02) **	7.19				
No High School	0.48	(0.23, 0.73) *	6.45	0.38	(0.17, 0.59) *	0.41	(0.21, 0.61) *
Age 65 and older	−0.29	(−0.56, −0.02) **	2.25	−0.19	(−0.47, 0.09)	−0.18	(−0.47, 0.11)
Age 17 and younger	−0.06	(−0.34, 0.22)	1.83	−0.15	(−0.42, 0.12)	−0.19	(−0.47, 0.09)
Disabilities	−0.20	(−0.47, 0.07)	3.13	−0.23	(−0.53, 0.07)	−0.19	(−0.48, 0.1)
Single parents	0.01	(−0.09, 0.11)	2.21	0.03	(−0.07, 0.13)	0.07	(−0.04, 0.18)
Minorities	−0.30	(−0.71, 0.11)	4.50	−0.43	(−0.76, −0.1) *	−0.40	(−0.73, −0.07) **
Limited English	−0.09	(−0.24, 0.06)	2.06	−0.10	(−0.26, 0.06)	−0.10	(−0.25, 0.05)
No vehicle	0.13	(0.08, 0.18) *	1.86	0.12	(0.06, 0.18) *	0.12	(0.06, 0.18) *
Biden voters	−0.10	(−0.19, −0.01) **	1.98	−0.10	(−0.19, −0.01) **	−0.11	(−0.20, −0.02) **
Population density	0.07	(0.02, 0.12) *	2.00	0.07	(0.01, 0.13) **	0.08	(0.02, 0.14) **
Adjusted R^2^	0.60			0.57		0.57	
Log likelihood	0.14			−3.23		−3.76	
Standard error	0.27			0.28		0.28	

Note: * *p* < 0.01; ** *p* < 0.05.

**Table 4:** Spatial regression results.

Variable	SAR		Durbin			
			Explanatory Variable		Spatial Lag	
	Coeff.	95% c.i.	Coeff.	95% c.i.	Coeff.	95% c.i.
Constant	4.07	(1.95, 6.19) *	−0.38	(−4.38, 3.62)		
Unemployment	0.01	(−0.19, 0.21)	−0.01	(−0.06, 0.04)	0.03	(−0.14, 0.2)
No High School	0.35	(0.14, 0.56) *	0.43	(0.22, 0.64) *	0.49	(0.05, 0.93) **
Age 65 and older	−0.22	(−0.48, 0.04) ***	−0.17	(−0.47, 0.13)	0.22	(−0.39, 0.83)
Age 17 and younger	−0.11	(−0.36, 0.14)	0.01	(−0.99, 1.01)	−0.08	(−0.67, 0.51)
Disabilities	−0.16	(−0.47, 0.15)	−0.06	(−0.39, 0.27)	0.08	(−0.72, 0.88)
Single parents	0.04	(−0.08, 0.16)	0.04	(−0.04, 0.12)	−0.06	(−0.37, 0.25)
Minorities	−0.37	(−0.7, −0.04) **	−0.51	(−0.89, −0.13) *	0.63	(−0.02, 1.28) ***
Limited English	−0.09	(−0.22, 0.04)	−0.08	(−0.23, 0.07)	0.01	(−0.99, 1.01)
No vehicle	0.12	(0.06, 0.18) *	0.11	(0.06, 0.16) *	−0.02	(−0.15, 0.11)
Biden voters	−0.13	(−0.22, −0.04) *	−0.20	(−0.3, −0.1) *	0.34	(0.08, 0.6) *
Population density	0.07	(0.01, 0.13) **	0.06	(−0.01, 0.13) ***	−0.07	(−0.18, 0.04)
*Wy*	0.32	(0.05, 0.59) **			0.85	(0.24, 1.46) *
Adjusted R^2^	0.65		0.66			
Log likelihood	−2.04		6.68			
Standard error	0.25		0.24			

Note: * *p* < 0.01; ** *p* < 0.05; *** *p* < 0.1.
